# Rejuvenating procholinergic treatments for cognition enhancement in AD: current challenges and future prospects

**DOI:** 10.3389/fnsys.2014.00254

**Published:** 2015-01-28

**Authors:** Brittney Yegla, Vinay Parikh

**Affiliations:** Department of Psychology and Neuroscience Program, Temple UniversityPhiladelphia, PA, USA

**Keywords:** acetylcholine, acetylcholinesterase (AChE) inhibitor, cognition, neurotrophins, Alzheimer's disease

Alzheimer's disease (AD) is an age-related neurodegenerative disorder characterized by global cognitive decline, with predominant impairments arising in attention and memory (Perry and Hodges, 1999). It is the sixth largest cause of death, and currently there is no way to prevent, cure, or even slow the progression (Klafki et al., 2006; Alzheimer's Association, 2014). Although there is a widespread decline in various neurotransmitter-containing cell bodies and axonal terminals in AD, the most consistent losses are seen in the basal forebrain (BF) cholinergic neurons and its projections (Mesulam, 2004; Schliebs and Arendt, 2011). Because of the documented role of the BF cholinergic system in learning and memory, the “cholinergic hypothesis” of AD was established (Bartus et al., 1982) and has been the primary directive for drug development and treatment in AD for almost three decades. While cholinomimetic drugs such as acetylcholinesterase (AChE) inhibitors, which elevate extracellular levels of the neurotransmitter acetylcholine (ACh), are the viable treatment option for AD and provide moderate alleviation to cognitive impairment, the magnitude of cognitive improvements with these drugs has remained limited (McGleenon et al., 1999; Raina et al., 2008). Additionally, these drugs are not successful in halting the progression of AD. Furthermore, the evidence that non-specific blockade of either muscarinic or nicotinic ACh receptors (mAChRs and nAChRs) alone produce dementia-like symptoms has remained inconsistent (Little et al., 1998; Erskine et al., 2004; Roegge and Levin, 2006). These issues have raised questions concerning the validity of the cholinergic hypothesis and whether the development of procholinergic therapies as cognition enhancers should be considered for AD. Here we argue that psychopharmacological approaches targeting the cholinergic system are based on previous conceptualizations of ACh regulating arousal states. We urge that emerging views from recent studies that refine our understanding of the cholinergic mediation of specific cognitive processes, and how cholinergic mechanisms interact with other pathological markers during the progression of AD, should be considered while designing procholinergic therapies. This discussion will also focus on the development of new drug candidates such as cholinergic receptor subtype-specific agonists, choline transporter (CHT) modulators and neurotrophin-based therapeutics to normalize cholinergic function in AD. Additionally, the need to combine multiple therapeutic approaches to slow AD progression and maximize cognitive benefits will be emphasized.

BF cholinergic neurons located in the medial septum, vertical and horizontal band of Broca, and nucleus basalis/substantia innominata complex innervate the cortical mantle, as well as the hippocampus. Traditionally, the BF cholinergic system was described as a diffusely organized neuromodulator system that influences information processing throughout the cortex and hippocampus in the awake brain and during REM sleep (Woolf, 1991). There are a plethora of neurophysiological studies that demonstrated that pairing BF stimulation with the stimulation of thalamic afferents enhanced the processing of sensory inputs, while the loss of cortical cholinergic inputs or administration of m/nAChR antagonists abolished this effect (Sarter et al., 2005). Additionally, a considerable amount of evidence generated from psychopharmacological and lesion studies indicated that the BF cholinergic system supports attentional functions, working memory, and memory consolidation (Furey et al., 2000; Power et al., 2003; Sarter et al., 2003). Furthermore, microdialysis studies illustrated performance-associated changes in ACh release in the cortex and hippocampus of rodents performing attention or memory tasks (Himmelheber et al., 2000; McIntyre et al., 2002). Together, these data suggested that the BF cholinergic system contributes to attention, learning, and mnemonic processes by generally inducing a state of arousal and elevating sensory processing by increasing the signal to noise ratio. Therefore, sustaining extracellular ACh release by terminating its highly efficient degradation process via AChE was considered a valid approach to restore cognitive function in AD.

AChE inhibitors have been in clinical practice to treat the cognitive symptomatology of mild to moderate AD for almost two decades. Tacrine was the first approved AChE inhibitor for AD. However, due to a faster half-life and potential to produce adverse effects, specifically liver toxicity, it was replaced by newer AChE inhibitors such as donepezil, galantamine and rivastigmine (Knapp et al., 1994; Ma et al., 2003; Di Santo et al., 2013). Although AChE inhibitors have been shown to improve cognitive, specifically attentional, functions in AD subjects (Foldi et al., 2005), these improvements are ultimately inadequate and new procholinergic approaches are needed (Raina et al., 2008; Pepeu and Giovannini, 2009). One possible explanation for limited therapeutic efficacy of AChE inhibitors might be that besides stimulating the postsynaptic cholinergic receptors, higher levels of baseline ACh levels at the cholinergic synapses may also stimulate presynaptic autoreceptors, such as muscarinic M2 receptors, which may shut down the recruitment of cholinergic inputs during information processing (Decossas et al., 2005). Furthermore, the behavioral consequences of sustained postsynaptic m/n AChR activation remain unknown. The uncoupling of presynaptic from postsynaptic cholinergic signaling is hypothesized to have profound effects on the neuromodulation of local and efferent circuitry limiting cognitive enhancement (Hasselmo and Sarter, 2011).

Advancements in understanding the multi-temporal modes of cholinergic transmission offer insight into developing drug treatments centered on cognition enhancement. The recent evolution of a biosensor-based electrochemical approach for monitoring cholinergic transmission in real time generated evidence that precisely orchestrated and temporally restricted changes in ACh release mediated specific cognitive operations. In task-performing animals, phasic (rapid; on a sub-second to second time scale) increases in cholinergic transmission in the prefrontal cortex mediated the detection of attention-demanding cues by switching perceptual processing of the cue to cue-evoked activation of response rules (Parikh et al., 2007; Howe et al., 2013). Such transient increases in behavior-evoked ACh release were not observed in the motor cortex, which was used as a neocortical control region. Moreover, performance-related tonic (slower; on the time scale of minutes) increases in ACh release, which occurred cortex-wide, fostered and maintained general readiness for input processing, and facilitated signal-driven processes required for learning and maintaining attention. The pattern of tonic changes in cholinergic transmission resembled performance-related cortical ACh release measured using microdialysis (Parikh and Sarter, 2008). These temporally-dissociated characteristics of ACh release patterns are also supported by the electrophysiological evidence demonstrating burst firing and tonic discharges of BF cholinergic neurons (Unal et al., 2012). Collectively, these studies led to a major revision of our view on ACh that was previously considered as a slowly releasing modulator of arousal augmenting the gain function of neurons, to now, as a neurotransmitter that encodes distinct cognitive operations. This view emphasizes a need to focus on designing cholinergic therapies targeting the phasic component of cholinergic transmission that is critical for signal detection.

Harnessing this updated view of cholinergic transmission, specific ligands that activate α4β 2 nAChRs may exert procognitive effects by amplifying cholinergic transients in AD subjects. Cortical cholinergic transients are generated based on local glutamatergic-cholinergic interactions (Sarter et al., 2009). Stimulus-driven recruitment of thalamocortical inputs increases glutamate release, which activates ionotropic glutamate receptors on cholinergic terminals and evokes phasic ACh release. These cholinergic transients foster detection of signals in attentional contexts, presumably by producing persistent spiking activity on cortical pyramidal neurons through postsynaptic mAChRs (Haj-Dahmane and Andrade, 1998). Activation of the high-affinity α4β 2 nAChRs residing on thalamocortical afferents also produces phasic increases in cholinergic activity via similar glutamatergic mechanisms (Parikh et al., 2008, 2010). A similar conceptual framework is applied to septo-hippocampal cholinergic circuits for encoding of episodic memories (Hasselmo and Sarter, 2011). Thus, α4β 2 nAChRs represent a valid biological target to develop procognitive drugs that act by facilitating phasic cholinergic signaling.

Another strategy would be to target cellular mechanisms that are involved in ACh production in cholinergic synapses and are critical to maintaining cholinergic transmission under conditions of higher cognitive load. The capacity to import choline into the presynaptic terminals via the high-affinity CHTs dictates the rate of ACh synthesis and release (Ferguson and Blakely, 2004; Sarter and Parikh, 2005). The mobilization of the intracellular pools of CHTs to the surface membrane (CHT trafficking) increases during attentional performance to maintain cholinergic transmission (Apparsundaram et al., 2005). Therefore, aberrations in CHT trafficking may influence phasic ACh release and attentional functions. In a recent study, we found that the capacity to generate cholinergic transients following sustained BF stimulation declined in CHT heterozygous mice (Parikh et al., 2013b). Moreover, these mutants displayed attentional impairments and disrupted trafficking of subcellular CHTs. These interesting results point toward an important role of CHT function in sustaining phasic cholinergic signaling to maintain cognitive functions. Given the evidence that high-affinity choline uptake declines in AD (Rodriguez-Puertas et al., 1994), future research on developing drugs that activate CHT function or increase the subcellular trafficking of CHTs holds promise to be a potential treatment for restoring cognitive deficits in AD.

An additional approach to developing procognitive therapies is targeting the interaction between BF cholinergic neurons, via n/m AChRs, and AD biomarkers. Among the neuropathological features of AD, the deposition of fibrillogenic β amyloid (Aβ) plaques and accumulation of intracellular neurofibrillary tangles containing hyperphosphorylated microtubule-associated protein tau are the two major hallmarks. Studies involving transgenic mice harboring mutations in AD-associated genes including amyloid precursor protein (APP), presenilin-1 and tau, have provided insights into possible reciprocal interactions between cholinergic markers and amyloidosis/tauopathy (Christensen et al., 2010; Perez et al., 2011). While it remains debated whether the cholinergic pathology is the primary cause or a consequence of AD, efforts to understand the relationship between cholinergic signaling and pathological substrates of AD are critical to understanding the etiology of AD. The interactions between Aβ and α7 nAChRs have remained complex. For example, the loss of α7 nAChRs produced cognitive decline and accumulation of soluble oligomeric forms of Aβ in 5-month old transgenic mice harboring the mutation for APP gene (Hernandez et al., 2010). Conversely, the activation of α7 nAChR with Aβ was shown to produce tau phosphorylation (Hu et al., 2008), and the deletion of this receptor gene improved memory impairments, reduced gliosis and preserved long-term potentiation in aged mice modeling the key pathological features of AD (Dziewczapolski et al., 2009). These data present a scenario where α7 nAChR agonists represent a potential strategy for controlling cognitive deficits in early AD that mostly result from synaptotoxic effects of Aβ oligomers, while blocking α7 nAChR function could alleviate cognitive symptoms during advanced stages of AD mostly associated with Aβ plaque and neurofibrillary tangles.

There is some evidence that mAChRs regulate APP processing. Specifically, the loss of M1 mAChRs has been shown to activate amyloidogenic processing of APP and greater accumulation of amyloid plaques in APP transgenic mice (Davis et al., 2010). Since M1 mAChRs are predominantly expressed in the cortex and hippocampus and play a major role in attention and memory (Soma et al., 2014), targeting these receptors as a therapeutic candidate for AD holds promise in compensating for cholinergic hypofunction while controlling Aβ. Currently efforts to develop positive allosteric modulators for M1 mAChRs as potential treatment for AD are ongoing.

Besides the role of Aβ and tau, oxidative stress and inflammation have also been considered to account for neurotoxicity in AD. It is important to note that some of the modest cognitive benefits of AChE inhibitors (above) have been actually ascribed to their anti-inflammatory properties, which involve inhibition of cytokine production and antioxidant effects (Chao, 2003; Tabet, 2006). Although a direct link between central cholinergic mechanisms and inflammatory processes is still lacking, more research in this area may provide new avenues to design procholinergic therapies for mitigating inflammation in AD.

The most significant obstacle in bolstering cholinergic and cognitive function in AD is the progressive loss of cholinergic innervation and neurons (Schliebs and Arendt, 2011). This has spurred drug discovery efforts to focus on developing neuroprotective therapies to preserve cholinergic function in AD. Nerve growth factor (NGF) is the primary neurotrophic factor supporting the growth, maintenance, and survival of BF cholinergic neurons by binding to the high-affinity tropomyosin-related kinase A (trkA) receptor (Fagan et al., 1997). Moreover, activation of p75 NGF receptors is known to exert detrimental effects on neurons by triggering apoptotic pathways (Chao, 2003). Postmortem studies have supported the notion that the loss of trkA receptors, and presumably the imbalance between trkA and p75 signaling, contributes to cholinergic dysfunction in AD (Mufson et al., 2008). We previously demonstrated that selective reduction of trkA receptors on BF cholinergic neurons produces persistent attentional impairments and decline in phasic cholinergic signaling in aged but not young rats (Parikh et al., 2013a). Moreover cholinergic dysfunction in trkA-suppressed aged rats occurred due to age-related accumulation of proNGF and overactivation of proNGF-p75 signaling (Yegla and Parikh, 2014). We also found that Aβ oligomers produced robust impairments in presynaptic cholinergic signaling and attentional capacities in aged rats (Parikh et al., 2014). Because Aβ oligomers are known to interact with the extracellular domain of p75 and produce neuritic degeneration in neuronal culture prepared from BF cholinergic neurons (Knowles et al., 2009), oligomeric Aβ-induced dysfunction of cholinergic synapses may be linked to p75 activation. Collectively, these findings support the view that interactions between aging/pathological aging and neurotrophic signaling escalate the vulnerability of the BF cholinergic system and neurotrophin-based therapies may have potential to rescue the loss of this neurotransmitter system in AD. Therefore, neuroprotective strategies that provide trophic support to cholinergic neurons or that restore trkA/p75 balance may offer advantage over the current drugs to halt cognitive deterioration in AD.

Recent research confirms that the era of developing a “magic bullet” to foster cognition enhancement in AD is over. Therefore, we need to consider devising strategies that focus on a more integrated or holistic therapeutic approach to preserve cholinergic function and halt cognitive deterioration in AD. In an ideal scenario, a combination of drugs that augment phasic cholinergic signaling, block the interactions between the pathological markers of AD and the cholinergic proteome, and activate neurotrophic signaling will maximize cognitive benefits.

## Conflict of interest statement

The authors declare that the research was conducted in the absence of any commercial or financial relationships that could be construed as a potential conflict of interest.
